# A Latent Class Analysis of Health Lifestyles in Relation to Suicidality among Adolescents in Mauritius

**DOI:** 10.3390/ijerph18136934

**Published:** 2021-06-28

**Authors:** Fanny Hoogstoel, Sékou Samadoulougou, Vincent Lorant, Fati Kirakoya-Samadoulougou

**Affiliations:** 1 Centre de Recherche en Epidémiologie, Biostatistique et Recherche Clinique, Ecole de Santé Publique, Université Libre de Bruxelles (ULB), Route de Lennik, 808, Bruxelles, 1070 Brussels, Belgium; fanny.hoogstoel@ulb.be; 2 Evaluation Platform on Obesity Prevention, Quebec Heart and Lung Institute, Québec City, QC G1V 4G5, Canada; ouindpanga-sekou.samadoulougou.1@ulaval.ca; 3 Centre for Research on Planning and Development (CRAD), Laval University, Québec City, QC G1V 0A6, Canada; 4 Institute of Health and Society (IRSS), Université Catholique de Louvain, 1200 Brussels, Belgium; vincent.lorant@uclouvain.be

**Keywords:** suicidality, suicidal behaviors, adolescents, latent class analysis, Mauritius

## Abstract

Suicidality, which includes suicidal thoughts, planning, and suicide attempts, results mainly from a combination of psychological, sociological, and environmental factors. Despite a high prevalence of suicidality among adolescents in Africa, only a few studies have considered these factors simultaneously. The objective of the study was to identify the prevalence of suicidality, to draw up profiles of concomitant risks, and to examine the associations between these profiles and suicidality in Mauritius. This study used data from the 2017 Mauritian Global School-based Student Health Survey including 3012 adolescents with a mean age of 14.9 ± 1.4 years. Factors related to lifestyle such as consumptions of alcohol and tobacco, physical activity, violence, parental support, anxiety, and loneliness were considered. A latent class analysis was performed to identify the profiles. Finally, a modified Poisson regression analysis with generalized estimating equations, adjusted with sociodemographic characteristics, was used to assess the association between these profiles and suicidality. Overall, more than one in ten adolescents had at least one of the suicidality behaviors. Three profiles were identified: 1 = “low risk group” (63.9%); 2 = “problems with violence” (15.2%); 3 = “problems with violence, alcohol, tobacco and psychological distress” (20.9%). Profiles 2 and 3 were mainly made up of males. Adolescents under 15 represented the majority of individuals in profile 2. Finally, the risk of suicidality was higher in adolescents belonging to profiles 2 and 3 compared to profile 1 for the three suicidality behaviors (profile 3: Prevalence ratio (PR) for suicidal thoughts = 1.26, 95% CI = 1.19–1.34; PR for planning = 1.23, 95% CI = 1.17–1.30; PR for attempt = 1.23, 95% CI = 1.17–1.29). This study highlights the high prevalence of suicidality and a list of concomitant risks, emphasizing this suicidality in Mauritian adolescents. Therefore, these results recommend focusing preventive efforts toward a simultaneous consideration of these factors.

## 1. Introduction

Suicide, a dramatic scourge in many parts of the world, kills nearly 800,000 people each year [1]. This is the third leading cause of death among people aged 15–19 years [1], so adolescents represent an important target to protect [2]. This high prevalence of suicidal behavior is especially important in low- and middle-income countries, particularly in Africa [3]. Countries such as Cape Verde, Cameroon, Ivory Coast, Equatorial Guinea, Eswatini, Lesotho, South Africa and Zimbabwe have the highest suicide rates in this region, with more than 10 cases per 100,000 people [4]. With devastating effects on the relatives of the victim and the significant social and economic costs for society [5], suicide has a significant impact on the populations of these regions.

Identifying the risk factors linked to suicide or suicidal tendencies is therefore a public health imperative. Recently, a study on the prevalence of suicidal behaviors and their mental risk factors among young adolescents in 46 low- and middle-income countries showed that factors associated with suicidal behaviors included being female and older age [3]. Moreover, many studies have shown that the transition from childhood to adolescence is characterized by the discovery and first use of alcohol and tobacco and other health risk factors. This period is also marked by other risky behaviors such as physical inactivity [6,7]. These factors may be associated with various future chronic diseases [8], a significant problem for our societies today. These adolescents, already exposed to these risks, are also more sensitive to multiple types of psychological distress that can extend into adulthood [9]. These typical adolescent behaviors are partly those associated with suicidal tendencies.

Indeed, individuals with a suicidal tendency are often prone to psychological distress problems, but may also be addicted to a psychotropic substance, more accustomed to violence or even more susceptible to loneliness [5]. Several studies have already looked at these factors associated with suicidality among adolescents [10,11,12]. However, these analyses considered these factors independently of one another. While some studies have shown associations between these behaviors (for example, between alcohol consumption and violence) [13], only a few analyses have been made on the clustering of these behaviors [14,15], and very few have studied their associations with suicidality among adolescents [16,17].

However, we now know that looking at multiple behaviors simultaneously helps to better focus health interventions [18], and it also leads to greater efficiency and lower costs [19]. Moreover, suicide prevention in adolescents requires screening strategies, such as the Teen Screen or the SEYLE programs, to identify those most at risk in order to provide these adolescents with appropriate services [20]. One possible method to study clusters is latent class analysis (LCA) [21]. LCA is a multivariate categorical data analysis technique. By classifying individuals from a heterogeneous population, this analysis provides the possibility of creating more homogeneous subgroups [22]. In particular, it makes it possible to identify and characterize clusters of similar cases and their proportions by getting closer to the distribution of observations through the variables and estimating the probability of each individual belonging to each subgroup [23,24].

Mauritius, also known as the Republic of Mauritius, is an island state in Africa, located in the Indian Ocean. Mauritius, a developing country, had 1,273,000 inhabitants in 2015 [25]. Mauritians have a life expectancy at birth of 71 years for men and 78 for women [25]. Mauritius has been an independent country since 1968 and has become a model of success over the years [26]. The economy of the country, which initially relied mainly on the export of sugar, now benefits from tourism and textile industries that allow it to continue its expansion [27]. Thus, Mauritius changed category in 2020, moving from a middle-income country to a high-income country [28,29]. Despite a positive political and economic context providing a good way of life, Mauritius, like many other countries, is affected by various mental illnesses and has difficulties in treating them [30]. The country’s many economic advances have led to considerable changes in lifestyle, especially among adolescents and several studies have thus been carried out on the behavior of adolescents and their consequences [31,32,33], but none have studied the association between these behaviors and suicidality.

Thus, the objectives of this study were to assess the prevalence of this suicidality; to identify different profiles from a list of risky behaviors such as violence, alcohol and tobacco consumption, or psychological distress among adolescents; to provide its sociodemographic characteristics, and to study the associations between these profiles and suicidality among adolescents in Mauritius.

## 2. Materials and Methods

### 2.1. Data Sources and Study Design

The data came from the Global School-based Student Health Survey (GSHS), developed by the WHO and carried out in Mauritius in 2017, which is, to date, the most recent data available on the lifestyle and suicidal behaviors of adolescents in Mauritius. The WHO survey aims to establish programs, health policies, and preventive interventions for the use and evaluation of school health, as well as health promotion of young people. This study allows countries and international agencies to make comparisons regarding the prevalence of health behaviors and protective factors [34].

With a high human development index (HDI) placing the country 64th [35], and more than 1.27 million inhabitants in 2015 [25], Mauritius is home to a considerable number of adolescents. In particular, this represented a total number of 94,895 adolescent boys and 91,977 adolescent girls in 2016 [36].

Among the 175 schools dispensing secondary general education in the country [37], schools were selected with a probability proportional to the size of the number of enrollments in each establishment. Classes were then selected at random, and all students in these groups were eligible to participate. Alcohol and tobacco consumption, eating behaviors, drug use, hygiene, mental health, physical activity, sexual behaviors, violence, and unintentional injuries were measured through computer-scanned responses. All selected schools participated, resulting in a 100% response rate from schools. Then, 84% of the students answered the questionnaires, which resulted in an overall response rate equal to 84%. The GSHS survey was approved by the Mauritius Ministry of Health. Participation was voluntary and all participants and their parents or guardians had given their consent.

In total, 3012 students from the 8th to 12th grades participated. Incomplete cases for this analysis represented 11% (335 individuals) of the adolescents.

### 2.2. Measures

The variables were selected on the basis of a literature review [12,17]. They were grouped into three groups of major determinants known to be linked to suicidality among adolescents: psychosocial distress, environmental factors and health risk behaviors. Sociodemographic characteristics (age, sex, grade, and socioeconomic status) were also noted for the remainder of the study. A summary of the original questions asked to the adolescents as well as the associated coding mechanisms, is presented in Appendix A Table A1.

#### 2.2.1. Psychosocial Distress

Psychosocial distress encompasses three criteria: anxiety, loneliness, and parental support. Anxiety was measured in response to the question “During the past 12 months, how often have you been so worried about something that you could not sleep at night?” Two categories of responses were then created: presence of anxiety and absence of anxiety. Loneliness was measured according to the same pattern with use of the following question “During the past 12 months, how often have you felt lonely?”

In order to assess social distress and therefore, the adolescents’ relationship with violence, the involvement in a physical fight and having been the victim of a physical attack were considered. An individual was considered not exposed to physical violence if they said that they had not been involved in a physical fight. The same pattern was used for the status of victims, with the answer obtained to the question “During the past 12 months, how many times were you physically attacked?”

#### 2.2.2. Environmental Factors 

In this study, parental support was considered an environmental factor. The associated variable was obtained according to the answer to the question “During the past 30 days, how often did your parents or guardians check to see if your homework was done?” If the answer was never, rarely or sometimes, the response was considered “unsupported.” Conversely, if the answer was most of the time or always, the variable took “presence of parental support” as a value.

#### 2.2.3. Health Risk Behaviors

The variable related to tobacco consumption was obtained following the answer to the question “During the past 30 days, on how many days did you smoke cigarettes?” The following responses were thus considered: No consumption or consumption. Alcohol consumption was measured in the same way, providing two groups of responses.

Finally, physical activity was measured over the last seven days preceding the questionnaire; an individual was considered active if they had been active for more than 60 min on at least five days.

#### 2.2.4. Suicidality

Suicidality was measured according to three criteria preceding the last 12 months before the questionnaire regarding whether the individual had considered suicide, planned how to do it, or made one or more suicide attempts. These criteria correspond respectively to the “consideration”, “planning”, and “attempt” variables, used later in the analysis.

#### 2.2.5. Sociodemographic Characteristics

The sociodemographic characteristics were age (11–15 years or 15 years and over [7,38]), gender (male or female), level of education (grades 8/9, grade 10, and grades 11/12) and socioeconomic status. Since the WHO’s data do not directly provide socioeconomic status, it was measured in terms of hunger over the past 30 days, as in the case of other similar studies [8,39]. Socioeconomic status was considered high if there was never or rarely a feeling of hunger and low if the answer to the question was sometimes, most of the time, or always.

### 2.3. Statistical Analysis

In total, 335 individuals were incomplete cases with at least one missing response. Multiple imputations were performed on adolescents with one missing value among the eight variables studied, while those with at least two missing values were excluded (58 individuals, 1.9% of the total population). For this, the predictive mean matching (PMM) method was applied using the “mice” package in R version 3.13.0.

#### 2.3.1. Descriptive Analysis

A descriptive analysis was carried out, taking into account the sampling weights and the sampling method (stratification and clustering). Proportions for each of the indicators were calculated, using the R “survey” package (version 4.0). 

The proportion of multiple behaviors was calculated. Each indicator was coded as follows: 0 = “no risk” and 1 = “presence of a risk”; the sum of the eight factors was calculated to obtain a value associated with multiple risks [7], with this value ranging from 0 (no risk) to 8 (presence of all risks).

##### 2.3.2. Latent Class Analysis (LCA) 

A latent class analysis was performed using R to identify and describe the classes associated with adolescents’ health risk behaviors. In order to determine the exact number of latent classes, an exploratory approach was used [17]. Starting from a two-class model, the analysis was carried out several times in a row, increasing this number of subgroups turn by turn and replicating each of the models 10 times for greater precision. 

Then, statistical model adjustment indices, such as the Bayesian information criterion (*BIC*), the adjusted *BIC*, the Akaike information criterion (*AIC*) [40], and the likelihood ratio, were used, coupled to the entropy values [41] and to the proportions of each of the classes to identify the final number of profiles. Indeed, the selected classes had to have enough observations to provide a representative profile of a population [42]. In practice, subgroups with a size of less than 5% were not retained, as in similar studies [17]. The poLCA package version 1.4.1 was used under R version 4.0.32.3.3. Modified Poisson Regression Analysis with Generalized Estimating Equations (GEEs)

To assess the association between profile membership and suicidality, a modified Poisson regression with generalized estimating equations was used [43]. Age, sex and socioeconomic status were also used as the adjustment covariates. This model allowed to deduce a prevalence ratio (PR) and its confidence interval. The significance level was set as 0.05 for this study. These analyses were carried out with the “geepack” package version 1.3–2) with R (version 4.0.3). 

## 3. Results

### 3.1. Sociodemographic and Behavioral Characteristics of Adolescents 

The sociodemographic characteristics of the study participants are presented in Table 1. Of the total sample, 62.3% of the total sample were between 11 and 15 years old. The mean age was 14.9 years with a standard deviation (*SD*) of 1.4. The male/female distribution was equitable, with 46.5% males and 53.5% females. The grade, which was separated into three categories, shows that 40.7% of adolescents were from grades 8 or 9, 23.5% from grade 10, and 38.5% from grades 11 or 12. Finally, only 24.3% of individuals had a high socioeconomic status. 

A total of 18.1% of the adolescents in Mauritius reported consuming tobacco with 26.6% and 6.2% consuming alcohol and marijuana, respectively. The majority had an insufficient level of physical activity, with 70.6% of them being considered inactive. The study of the report on violence showed that 23.0% had previously been physically attacked and 29.0% had been involved in a physical fight. Analysis of indicators associated with psychological distress and environmental factors showed that only 32.1% had sufficient parental support, 9.4% had anxiety disorders, and 10.7% were affected by loneliness.

**Figure 1:**
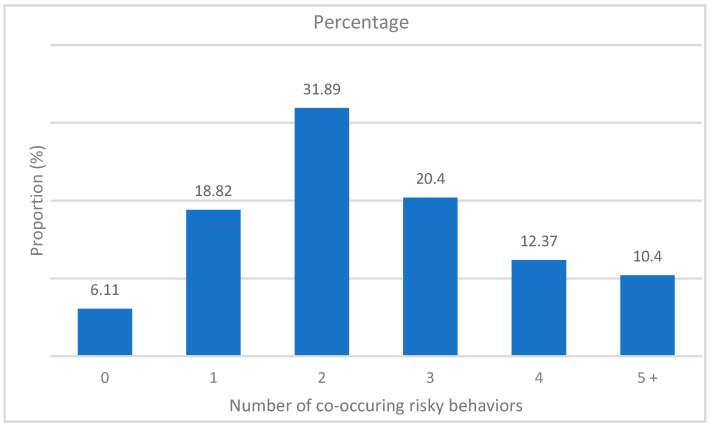
Prevalence of the number of co-occurring risky behaviors among adolescents aged 11–18 in Mauritius, 2017.

Regarding the concomitance of these factors, Figure 1 shows the prevalence of each level of the “multirisk” variable, corresponding to the sum of the cumulative individual risky behaviors. For example, more than four in ten adolescents cumulated at least three risks simultaneously.

### 3.2. Adolescent Suicidality

Table 2 presents the prevalence of each of the factors associated with suicidality according to sociodemographic characteristics. If we consider suicidal tendencies as having considered suicide, 16.1% (95% CI = 13.4–18.7) of the adolescents were concerned. This result was higher in females, with a prevalence of 19.4% (95% CI = 15.7–24.0). The youngest were also more likely to consider suicide, with a prevalence of 16.6% (95% CI = 11.4–20.0) among those aged 11–15 years. This was confirmed by these percentages according to grades: 18.1% of adolescents from grades 8 or 9 had previously considered suicide. Finally, adolescents with a low socioeconomic status tended to be more prone to this suicidality, with a prevalence of 22.3% (95% CI = 17.7–28.0) against 14.1% (95% CI = 11.6–17.0) for those with a high socioeconomic status.

The prevalence of suicide planning was somewhat low (14.7%, 95% CI = 12.8–17.0). As for the “consideration” factor, females and adolescents with a low socioeconomic status were factors accentuating the possibility of thinking about how to attempt suicide. On the contrary, young age was, this time, characteristic of a lower prevalence (14.1%, 95% CI = 12.1–16.0); adolescents over 15 years old were more likely to report planning (15.5%, 95% CI = 13.0–18.0).

The prevalence of suicide attempts was the lowest (12.8%, 95% CI = 10.8–15.0). Again, females had a higher prevalence of suicide attempts than males. Younger people and adolescents with a low socioeconomic status were also characteristic of a higher prevalence.

If we take a closer look at this suicidality, 231 adolescents (7.7% of the total population) declared having attempted suicide once, 82 having had two or three attempts (3.0%), 27 having had four or five attempts (0.9%) and 27 having had six or more attempts (1.2%) [44].

Finally, among the 3012 students who participated in the survey, 177 adolescents combined the three factors associated with suicidality (consideration, planning, and attempt); 240 adolescents considered suicide and attempted suicide while 201 individuals planned and attempted suicide.

### 3.3. Identification of Profiles and Associated Characteristics

#### 3.3.1. Model Choice by Latent Class Analysis

The results obtained from the latent class analysis are presented in Table 3. The *BIC* values showed a minimum for a five-class distribution. However, from a division into four profiles, some proportions of distributions fell below the 5% limit threshold. Therefore, by coupling the values of *BIC*, *AIC*, percentages of proportions and entropy, a model with three latent classes was retained. With the highest possible entropy value, equal to 0.76, this model allowed for better distinction between profiles [45].

#### 3.3.2. Characteristics of the Identified Profiles

The characteristics specific to each class are presented in Table 4. Among the three profiles obtained, the first one was defined by adolescents presenting the least risk behaviors (“low-risk group”), representing 63.9% of the total sample. Profile 2 (“problem with violence”) included 15.2% of the adolescents. These were adolescents with high-risk behavior in relation to violence. Indeed, the proportions representing involvement in a physical fight and having been physically attacked were equal to 100% (against 0.0% in profile 1) and 40.2% (against 11.0% in profile 1), respectively. Profile 3 (“problems with violence, alcohol, tobacco, and psychological distress”) was characterized by adolescents with problems of violence, but also of tobacco and alcohol consumption, with proportions equal to 67.6% (against 4.1% in profile 1 and 4.6% in profile 2) and 84.1% (against 12.5% in profile 1 and 0.0% in profile 2), respectively. Psychological distress, represented by anxiety and loneliness in this study, was also a particular characteristic of profile 3, with a large increase in the proportion associated. A total of 20.9% of adolescents were included in profile 3.

Inactivity and lack of parental support were two indicators with relatively high proportions for each of the profiles.

The top of Table 4 presents the sociodemographic characteristics of each of the profiles. Females mainly made up profile 1, while males were predominantly focused in profiles 2 and 3. The youngest (those aged 11–15 years and in grades 8–9) were particularly sensitive to violent behavior, representing the majority of the adolescents included in profile 2. The mean age of the adolescents making up profile 1 was 14.9 years (*SD* = 1.38), in profile 2 was 14.3 years (*SD* = 1.29), and in profile 3 was 15.2 years (*SD* = 1.40). Few differences in socioeconomic status were observed except for a slight increase in the proportion of individuals with low socioeconomic status in profile 3 compared to the first two profiles.

### 3.4. Risky Behaviors and Suicidality

The results obtained from the modified Poisson regression with generalized estimation equations are presented in Table 5. A significant association between suicidal thoughts (“consideration”) and belonging to profiles 2 and 3 (relative to profile 1) was shown. Adjusted for age, sex and socioeconomic status, the adolescents in profile 2 (*PR* = 1.07, 95% CI = 1.03–1.11) and those in profile 3 (*PR* = 1.26, 95% CI = 1.19–1.34) had a higher proportion of suicidal tendencies than the adolescents in the low-risk group.

The same associations were found for the “planning” factor: The adolescents in profiles 2 and 3 were more likely to have planned a suicide attempt (with *PR* = 1.04, 95% CI = 1.00–1.07 and *PR* = 1.23, 95% CI = 1.17–1.30, respectively) compared to those in profile 1. 

Finally, and still after adjusting for sociodemographic characteristics, the same significant associations were shown between having made at least one suicide attempt and belonging to profiles 2 and 3, with prevalence ratios equal to 1.06 (95% CI = 1.02–1.10) and 1.23 (95% CI = 1.17–1.29), respectively.

## 4. Discussion

The objective of the study was to identify the prevalence of suicidality, to draw up profiles of concomitant risks, and to examine the associations between these profiles and suicidality in Mauritius.

First, this analysis showed that around 16% of adolescents had suicidal thoughts during the 12 months preceding the study, that more than one in ten adolescents had previously thought about the means they would use, and that 13% had previously attempted suicide at least once. Females were more concerned by these aspects of suicidality: 1 in 5 adolescent females answered positively to the question related to suicidal thoughts and 17% and 14% had previously planned or attempted suicide, respectively. Fortunately, Mauritian adolescents with suicidal thoughts outnumbered those who had actually attempted suicide. This observation was expected [46,47], but this difference in prevalence represents a significant risk for the future mental health of these adolescents. Indeed, this rather high percentage of suicidal thoughts can lead to real traumas in future adulthood. Recent studies have shown that suicidality and associated behaviors during childhood and adolescence are a robust risk of re-observing these trends in adulthood [48]; adults who had suicidal tendencies in adolescence are more likely to repeat these behaviors than those who never had suicidal thoughts [49].

Second, this study made it possible to identify several concomitant health behaviors, thus creating three distinct profiles. Finally, it was demonstrated that belonging to groups with multiple risky behaviors is associated with a greater risk of suicidality, regardless of the factor of suicidality studied. Thus, violence, tobacco use, and alcohol use were, when observed concomitantly, factors associated with an increased risk of suicidality, as were loneliness and anxiety.

### 4.1. Profiles Obtained and Suicidality

Latent class analysis yielded three profiles. The first, grouped together with the least risky behaviors for health, concerned more than three in five adolescents. Profile 2, representing 15% of the individuals in this study, was mainly associated with a problem of violence. The third profile grouped together the riskiest behaviors and concerned one in five adolescents. In this latter group, tobacco and alcohol consumption added to the problems of violence, which was a clustering also observed in Kenya, for example [50]. These smoking and alcohol behaviors are known as predisposing factors for injury and violence [51]. This last profile also showed the concomitant use of tobacco and alcohol, substances frequently used together [7,19]. The adolescents in profile 3 were also more susceptible to anxiety and loneliness. It has been shown that the consumption of alcohol and tobacco reinforces violence and psychological distress [52,53].

Finally, differences in sociodemographic characteristics were observed between each of the profiles. Profile 1, the “low-risk group,” mainly composed of adolescent females with a high socioeconomic status, was characteristic of “good” health behaviors. On the contrary, males mostly made up profile 2, “problems with violence,” and 3, “problems with violence, alcohol, tobacco, and psychological distress.” Age was also an important sociodemographic characteristic: Older adolescents were more likely to have multiple risky behaviors and, therefore, to belong to profile 3 compared to profiles 1 and 2. This association between sex, age, and membership in higher risk groups has already been observed in several studies [19,50,54,55].

Now, if we look at the association between these profiles and suicidality, this study showed that the adolescents in profiles 2 and 3 had an increased risk of suicidality, regardless of the factor studied (consideration, planning, or attempt). This increase in probability was associated with the following factors: the consumption of tobacco and alcohol or the tendency of violence, observed concomitantly. These results have been found in numerous studies based on data from the GSHS survey in Africa [12,39,56]. Anxiety and loneliness, factors associated with so-called psychological distress, were also indicators associated with the increased likelihood of suicide. These results have also been found in other African countries, such as Mozambique [57].

This study also showed that approximately one in four adolescents who took part in this survey declared having consumed alcohol at least once in the 30 days preceding the distribution of the questionnaire. Likewise, one in five adolescents declared having used tobacco. With a civilian majority set at 18, it would seem that these adolescents still had access to these substances, which represent a particular danger when they are combined [58,59]. However, it is not surprising to find a high tendency toward alcohol consumption among these young Mauritians. Previously, in 2013, serious cases of alcoholism were detected in a relatively young population, occupying a large part of the issue managed by the country’s centers [60].

Finally, physical inactivity was a risky health behavior found in each of the profiles observed. A known and common problem in most low- or moderate-income countries, especially in Africa [61], as several studies have previously shown, is the existence of associations between a sedentary lifestyle and suicidality [62,63,64].

### 4.2. Implications for Public Health 

This study makes it possible to establish several recommendations in terms of public health. First, this analysis showed the importance of taking into account factor groupings in health interventions. Indeed, with an association between the profiles obtained and suicidality having been found, studying only the factors individually seems no longer sufficient if we are interested in the state of health of these adolescents. In profile 3, “problems with violence, alcohol, tobacco, and psychological distress,” a larger number of health risk behaviors were observed simultaneously. It is therefore necessary to tackle these problems in the same approach in order to reach the affected individuals as much as possible. Likewise, differences in sociodemographic characteristics were observed between profiles. These characteristics are also to be taken into account; for example, adolescent females are not affected in the same way as males by suicidality.

This study also provided information on the classification of adolescents. Indeed, latent class analysis is a technique that identifies and quantifies inter-individual variability of the health indicators used [65]. This makes it possible to establish a number of profiles that can explain the associations between the different behaviors studied. This analysis could therefore be used whenever one wishes to classify and study a population of adolescents. By using latent class analysis, future studies could therefore also help organizations, health policymakers, or even administrations to better understand the different profiles observed in adolescents and thus make it possible to better target interventions, for example, by having a better distribution of resources.

### 4.3. Strengths and Limitations 

The results of this analysis should be interpreted by taking into account the limitations.

First, since this was a cross-sectional study, this analysis does not make it possible to establish causal links.

Second, the data used in this study came from self-reports of the participating adolescents. Some may have underestimated or, on the contrary, overestimated some of their behaviors, causing a desirability bias. For some questions, the adolescents may also have been unwilling to answer due to social or family pressures, causing a non-response bias. In addition, the questions asked to these adolescents used different deadlines (30 days, 12 months, etc.); this could lead to a memory bias.

In addition, the variables used in this study were mostly categorized only in a binary pattern and only two possible options were given. This was the pattern chosen for each of these indicators by the WHO during the GSHS survey. However, when looking at tobacco or alcohol consumption, occasional consumption has been associated with frequent consumption; the distinction between the two was therefore not made. Likewise, the measures of anxiety and loneliness did not make it possible to study several dimensions: only 2 categories (presence/absence) were made. However, recent studies have developed new methods of measuring anxiety. For example, the GAD-7 scale, scored from 0 to 21 to assess the Generalized Anxiety Disorder (GAD), allows the creation of several dimensions [66]. The Revised Child Anxiety and Depression Scale (RCADS) is another possible measure that takes into account a greater number of dimensions to assess anxiety and depression. With 47 items, this self-reported questionnaire [67] could, as the GAD-7, be a means of better understanding the psychological distress of these adolescents.

This study did not use certain factors that are known to have associations with suicidality, such as insufficient sleep time or too much screen time [17]; these data were not collected by the WHO for Mauritius.

Finally, despite the large number of schools included in the study, the results obtained cannot be generalized to all adolescents in Mauritius. In fact, data from the Global School-based Student Health Survey (GSHS) did not allow the inclusion of out-of-school adolescents, despite representing almost 30% of individuals aged 12–19 in 2019 [68] and being more likely to report health risk behaviors [69].

However, this study was the first to explore the clustering of risky behaviors for health among adolescents in Mauritius. It notably used latent class analysis, a robust and person-centered approach to identify adolescent profiles over a wide range of samples. This study also benefited from a relatively high response rate from Mauritian adolescents.

## 5. Conclusions

In conclusion, this study showed the high prevalence of suicidality in Mauritius and for the first time, made it possible to identify the profiles of health-related behaviors associated with suicidality.

These associations underline the importance of taking into account these concomitant risk profiles in health interventions aimed at preventing suicidality among adolescents in Mauritius.

## Figures and Tables

**Table 1:** Sociodemographic and behavioral characteristics of adolescents aged 11–18 in Mauritius, 2017.

Sociodemographic Characteristics	Frequency	%
Age (mean ± SD: 14.9 ± 1.4)		
11–15	1957	62.3%
>15	1052	37.7%
Sex		
Male	1414	46.5%
Female	1584	53.5%
Grade		
8–9	1196	40.7%
10	807	23.5%
11–12	976	35.8%
Socioeconomic status		
High	712	24.3%
Low	2262	75.7%
**Psychosocial distress**		
Anxiety	283	9.4%
Loneliness	313	10.7%
Frequently physically attacked	685	23.0%
Frequently involved in a physical fight	863	29.0%
**Environmental factors**		
Lack of parental support	955	32.1%
**Health risk behaviors**		
Frequent tobacco consumption	507	18.1%
Frequent alcohol consumption	755	26.6%
Inactive	2079	70.6%

Notes: SD, Standard Deviation.

**Table 2:** Prevalence of suicidality among adolescents aged 11–18 in Mauritius, 2017.

	Consideration	Planning	Attempt
	**Prevalence % (95% CI)**	**Prevalence % (95% CI)**	**Prevalence % (95% CI)**
**Total**	16.1 (13.4–18.7)	14.7 (12.8–17.0)	12.8 (10.8–15.0)
**Age**			
11–15	16.6 (13.7–20.0)	14.1 (12.1–16.0)	14.3 (11.9–17.0)
>15	15.1 (11.4–20.0)	15.5 (13.0–18.0)	10.4 (7.8–14.0)
**Sex**			
Male	12.2 (10.2–15.0)	11.2 (9.9–14.0)	10.8 (8.6–14.0)
Female	19.4 (15.7–24.0)	17.4 (15.1–20.0)	14.4 (11.5–18.0)
**Grade**			
8–9	18.1 (13.9–23.0)	14.2 (11.4–17.0)	14.6 (10.8–19.0)
10	14.7 (9.9–20.0)	14 (10.7–18.0)	12.76 (9.9–16.0)
11–12	15.4 (13.0–18.0)	15.5 (13.1–18.0)	10.73 (8.0–14.0)
**Socioeconomic status**			
Low	22.3 (17.7–28.0)	20.4 (17.6–23.0)	17.6 (14.0–22.0)
High	14.1 (11.6–17.0)	12.8 (10.9–15.0)	11.27 (9.2–14.0)

Notes: 95% CI—95% confidence interval.

**Table 3:** Latent class analysis results for the identification of the number of profiles among adolescents aged 11–18 in Mauritius, 2017.

Number of Classes	*BIC*	*aBIC*	*cAIC*	*LR*	Class 1	Class 2	Class 3	Class 4	Class 5	Class 6	Entropy
1	23,996.00	23,970.58	24,004.00	1515.79	100%	-	-	-	-	-	-
2	23,226.18	23,172.17	23,243.18	674.05	71.7%	28.3%	-	-	-	-	0.53
3	23,143.30	23,060.69	23,169.30	519.25	64.0%	20.6%	15.4%	-	-	-	0.76
4	23,076.94	22,965.73	23,111.94	380.97	3.6%	71.9%	15.3%	9.2%	-	-	0.62
5	23,050.60	22,910.79	23,094.60	282.71	3.5%	57.9%	11.4%	11.7%	15.5%	-	0.55
6	23,076.78	22,908.38	23,129.78	236.98	11.9%	2.8%	15.2%	3.6%	51.8%	14.7%	0.54

Notes: *BIC—*Bayesian Information Criterion; *aBIC—*adjusted *BIC*; *cAIC—*consistent Akaike Information Criterion; *LR*—Likelihood Ratio.

**Table 4:** Characteristics of the profiles obtained in adolescents aged 11–18 in Mauritius, 2017.

	Profile 1	Profile 2	Profile 3
Total Proportion	63.9%	15.2%	20.9%
**Sociodemographic characteristics**			
Age			
11–15	60.9%	79.3%	53.5%
>15	39.1%	20.7%	46.5%
Sex			
Male	37.4%	65.8%	58.4%
Female	62.6%	34.2%	41.6%
Grade			
Grade 8–9	37.6%	60.2%	34.8%
Grade 10	22.9%	20.7%	27.2%
Grade 11–12	39.5%	19.1%	38.0%
Socioeconomic status			
Low	21.4%	26.4%	31.4%
High	78.6%	73.6%	68.6%
**Psychosocial distress**			
Anxiety	6.6%	4.9%	20.8%
Loneliness	7.5%	7.8%	21.9%
Frequently physically attacked	11.0%	40.2%	45.1%
Frequently involved in a physical fight	0.0%	100.0%	63.2%
**Environmental factors**			
Lack of parental support	34.1%	37.3%	21.6%
**Health risk behaviors**			
Frequent tobacco consumption	4.1%	4.6%	67.6%
Frequent alcohol consumption	12.5%	0.0%	84.1%
Inactive	72.3%	62.9%	70.4%

**Table 5:** Association between suicidality and profiles in adolescents aged 11–18 in Mauritius, 2017.

Suicidality			
	Consideration *PR* (95% CI)	Planning *PR* (95% CI)	Attempt *PR* (95% CI)
**Profiles (reference: Profile 1, low-risk group)**			
Profile 2 (problems with violence)	1.07 (1.03–1.11) ***	1.04 (1.00–1.07) *	1.06 (1.02–1.10) **
Profile 3 (problems with violence, alcohol, tobacco, and psychological distress)	1.26 (1.19–1.34) ***	1.23 (1.17–1.30) ***	1.23 (1.17–1.29) ***
**Sociodemographic characteristics**			
Sex (reference: Male)			
Female	1.11 (1.07–1.15) ***	1.09 (1.06–1.13) ***	1.07 (1.04–1.10) ***
Age (reference: 11–15)			
>15	0.98 (0.95–1.02)	1.01 (0.98–1.03)	0.96 (0.93–0.98) ***
Socioeconomic status (reference: low)			
High socioeconomic status	0.94 (0.91–0.97) ***	0.94 (0.91–0.97) ***	0.96 (0.93–0.98) **

Notes: *PR—*prevalence ratio; 95% CI—95% confidence interval; *: *p* < 0.05; **: *p* < 0.01; ***: *p* < 0.001.

## Data Availability

Data for this study was obtained from the World Health Organization (WHO) website, is freely available online and can be downloaded (https://extranet.who.int/ncdsmicrodata/index.php/catalog/669/get_microdata, accessed on 19 May 2021).

## Appendix A

**Table A1:** Measures used for this study.

Indicators	Question	Type
**Sociodemographic status**		
Age	How old are you?	11–15/>15
Sex	What is your sex?	Male/Female
Grade	In what grade are you?	8–9/10/11–12
Socioeconomic status	During the past 30 days, how often did you go hungry because there was not enough food in your home?	Most of the time, Always/Sometimes, Rarely, Never
**Psychosocial distress**		
Anxiety	During the past 12 months, how often have you been so worried about something that you could not sleep at night?	Most of the time, Always/Sometimes, Rarely, Never
Loneliness	During the past 12 months, how often have you felt lonely?	Most of the time, Always/Sometimes, Rarely, Never
Physically attacked	During the past 12 months, how many times were you physically attacked?	1/2–3/4–5/6–7/8–9/10–11/>12
Involved in a physical fight	During the past 12 months, how many times were you in a physical fight?	1/2–3/4–5/6–7/8–9/10–11/>12
**Environmental factors**		
Parental support	During the past 30 days, how often did your parents or guardians check to see if your homework was done?	Most of the time, Always/Sometimes, Rarely, Never
**Health risk behaviors**		
Tobacco	During the past 30 days, on how many days did you smoke cigarettes?	0/1/2–3/3–5/6–9/10–19/20–29/30
Alcohol	During the past 30 days, on how many days did you have at least one drink containing alcohol?	0/1/2–3/3–5/6–9/10–19/20–29/30
Physical activity	During the past 7 days, on how many days were you physically active for a total of at least 60 min per day?	0 1 2 3 4/5 6 7
**Suicidality**		
Consideration	During the past 12 months, did you ever seriously consider attempting suicide?	Yes/No
Planning	During the past 12 months, did you make a plan about how you would attempt suicide?	Yes/No
Attempt	During the past 12 months, how many times did you actually attempt suicide?	0/1/2-3/4–5/>6
